# T Cell Receptor Mediated Calcium Entry Requires Alternatively Spliced Ca_v_1.1 Channels

**DOI:** 10.1371/journal.pone.0147379

**Published:** 2016-01-27

**Authors:** Didi Matza, Abdallah Badou, Kathryn G. Klemic, Judith Stein, Usha Govindarajulu, Monica J. Nadler, Jean-Pierre Kinet, Amnon Peled, Oz M. Shapira, Leonard K. Kaczmarek, Richard A. Flavell

**Affiliations:** 1 Department of Natural Sciences, The Open University of Israel, 1 University Road, Ra’anana, 4353701, Israel; 2 Genetic and Molecular Pathology Laboratory, Casablanca School of Medicine and Pharmacy, Hassan II University, Casablanca, Morocco; 3 Virginia Tech Applied Research Corporation, 900 North Glebe Road, Arlington, VA, 22203, United States of America; 4 Department of Immunobiology, Yale University School of Medicine, 300 Cedar street, New Haven, CT, 06520, United States of America; 5 Howard Hughes Medical Institute, Yale University School of Medicine, New Haven, CT, 06520, United States of America; 6 Yale Center for Clinical Investigation, Yale University School of Medicine, New Haven, CT, 06520, United States of America; 7 Department of Pathology, Beth Israel Deaconess Medical center, Boston, MA, 02215, United States of America; 8 Goldyne Savad Institute of Gene Therapy, Hadassah University Hospital, Jerusalem, 91120, Israel; 9 Department of Cardiothoracic Surgery, Hadassah University Hospital, Jerusalem, 91120, Israel; 10 Department of Pharmacology, Yale University School of Medicine, New Haven, CT, 06520, United States of America; 11 Department of Cellular and Molecular Physiology, Yale University School of Medicine, New Haven, CT, 06520, United States of America; University of Houston, UNITED STATES

## Abstract

The process of calcium entry in T cells is a multichannel and multi-step process. We have studied the requirement for L-type calcium channels (Ca_v_1.1) α1S subunits during calcium entry after TCR stimulation. High expression levels of Ca_v_1.1 channels were detected in activated T cells. Sequencing and cloning of Ca_v_1.1 channel cDNA from T cells revealed that a single splice variant is expressed. This variant lacks exon 29, which encodes the linker region adjacent to the voltage sensor, but contains five new N-terminal exons that substitute for exons 1 and 2, which are found in the Ca_v_1.1 muscle counterpart. Overexpression studies using cloned T cell Ca_v_1.1 in 293HEK cells (that lack TCR) suggest that the gating of these channels was altered. Knockdown of Ca_v_1.1 channels in T cells abrogated calcium entry after TCR stimulation, suggesting that Ca_v_1.1 channels are controlled by TCR signaling.

## Introduction

Calcium ion entry across the plasma membrane is necessary for the initiation of T lymphocyte activation and proliferation following antigen encounter [1–5]. A typical calcium response occurs in two distinct steps. Initially, calcium is released from the intracellular stores, like the ER [6], which then triggers extracellular calcium entry through store-operated calcium (SOC) channels in the plasma membrane [7, 8]. Activation of NFAT occurs upon elevation in cytosolic free calcium levels, which results in its retention in the nucleus and subsequent gene transcription [9, 10]. This process is modulated by variations in the amplitude and/or duration of the calcium signal [11], which subsequently affect gene transcription and thus T cell activation and differentiation.

Apparently, a wide variety of calcium channels participate in calcium entry to T lymphocytes [12, 13]. The most studied pathway for calcium entry in non-excitable cells is the CRAC (Calcium Release Activated Calcium Channel) pathway and its two key players, the stromal interaction molecule 1 (STIM1) and ORAI1 (also known as CRACM1 or TMEM142A) (reviewed in [14–16]). However, recent reports using deletion of ORAI or STIM proteins suggest that there are other pathways of calcium entry and that other plasma membrane calcium channels might be functionally involved [17–19].

Voltage gated calcium channels are known to mediate calcium entry in excitable cells [20]. The Ca_v_ channel complex contains the pore-forming α1 subunit and the auxiliary subunits α2, δ, γ, and β subunits, which play a critical regulatory role [20]. A total of ten α_1_ subunits have been identified and divided into 5 groups (L, P or Q, N, R, T) based on their properties [20]. The α_1_ subunit compose (~190 kDa in molecular mass) the actual functional calcium selective pore. It is composed of four homologous domains (I–IV) each containing six transmembrane α-helices (S1–S6). The α_1_ subunit also contains the voltage-sensing machinery (composed of the S4 helix from each domain). These channels are subject to rapid inactivation, which consist of two components: voltage-dependent (VDI) and calcium-dependent (CDI) [21]. The latter is mediated by the binding of calmodulin (CaM) to the channel [21].

Growing evidence suggests that these channels also contribute to calcium entry in non-excitable cells. In fact, several studies have suggested the functional presence of Ca_v_ channels in T lymphocytes (a non-excitable cell type), using pharmacological approaches [22–26]. We have examined the role of Ca_v_ channels and associated proteins in T cells. We have shown that CD4^+^ T cells express α1 subunits of the Ca_v_1 calcium channel family, but not Ca_v_2 or Ca_v_3 [27] and demonstrated the importance of the Ca_v_ β3 and β4 regulatory subunits in TCR-triggered calcium response, NFAT nuclear translocation, and cytokine production [27–29]. More recently, we have demonstrated the importance of a scaffold protein AHNAK1 in regulating calcium signaling in peripheral CD4^+^ T cells. AHNAK1 is associated with the regulatory β2 subunit of Ca_v_ channels and is required for normal expression of the Ca_v_1.1 α1 subunit and calcium influx after TCR cross-linking in CD4^+^ T cells [30].

T cells from both β4 or AHNAK1 deficient mice (the latter was generated in our lab [31]) have reduced Ca_v_1.1 channel membrane expression, deficient calcium entry and IL-2 production [27, 30]. Altogether, these observations suggested that the Ca_v_1.1 channels are functionally active in T cells in vivo.

An unresolved question is how are these T cell Ca_v_1.1 channels gated? Several studies, including ours, have shown that in contrast to excitable cells, treatment of T cells with KCl, to induce an artificial depolarization, does not lead to calcium entry [26, 27, 32]. While Ca_v_1.1 channels are likely functional in T cells, Ca_v_1.2 channels are apparently inactivated by direct interaction with STIM1 [33, 34]. In addition, another recent report suggests naïve CD44low T cells may have calcium currents dependent on Ca_v_1.4 channels, possibly due to their inherent conductance in the hyper-polarized range (i.e. at/near T cell resting membrane potentials) [35]. Therefore unique subtleties for Ca_v_ α1 subunit activation and gating appear to exist in T cells and possibly other non-excitable cell types.

With the functional effects of β3 and β4 Ca_v_ accessory subunits and Ca_v_1.4 channels already demonstrated in T cells [27, 28, 30, 35], we hypothesized that Ca_v_1.1 channels may be active in T cells, but with electrophysiological properties distinct from those expressed by excitable cells. In this paper, we report the sequence of Ca_v_1.1 channels in T cells and show that calcium entry after TCR stimulation is dependent on these channels. Together with prior studies [22–26, 36], these data suggest a physiological function for Ca_v_1.1 channels in T lymphocytes.

## Results

### Expression of Ca_v_1.1 in Activated T Cells

**Fig 1:**
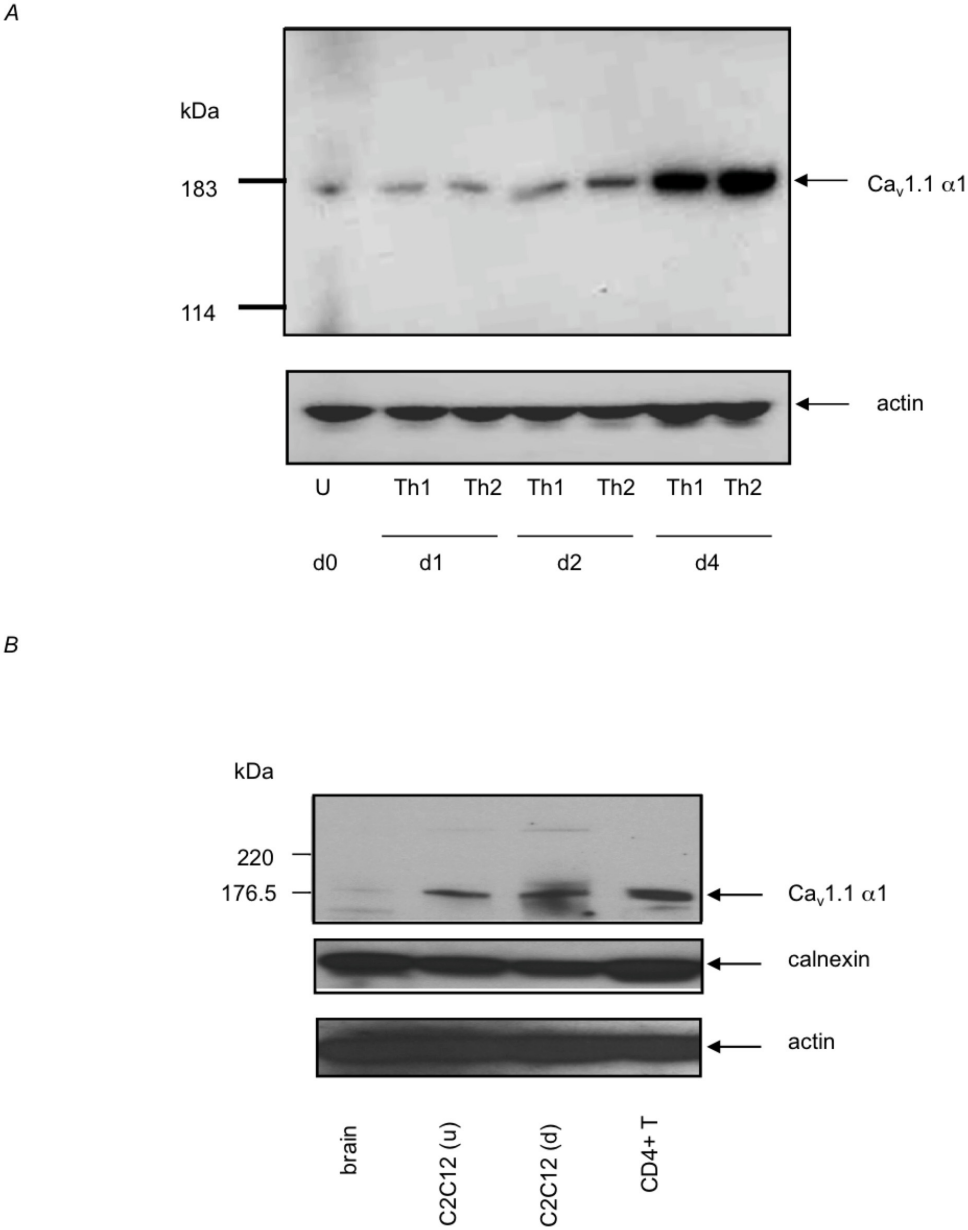
Ca_v_1.1 channel protein expression in CD4 T cells after stimulation. (A) Purified unstimulated (day 0) naïve CD4 T cells were stimulated with plate-bound antibodies to CD3 and CD28 under either Th1 or Th2 conditions (see experimental procedures for detail) for the indicated period of time. Immunoblot detection of the Ca_v_1.1 channel is shown. Actin was used as loading control. (B) Protein extracts from brain, undifferentiated (u) or differentiated (d) C2C12 skeletal muscle cell line, and from CD4 T cells were analyzed for the expression of Ca_v_1.1 channels. Calnexin and actin were used as internal controls. Results are representative of at least three experiments.

The expression of Ca_v_ channels mRNA was previously described in primary T cells [27]. During primary stimulation of T cells, Ca_v_1.1 channels mRNA is constitutively expressed [30]. Therefore, we examined the expression of Ca_v_1.1 channel protein in primary and activated T cells (Fig 1A). Using immunoblot analysis, we found that Ca_v_1.1 channel protein is detected in undifferentiated primary CD4^+^ T cells, and is up-regulated in differentiated effector Th1 (incubated with IL-12 plus anti-IL-4) and Th2 (incubated with IL-4 plus anti-IFNγ) cells (Fig 1A). Ca_v_1.1 channels expression level and protein molecular weight were compared to other cell types by immunoblotting using protein extracts from brain (as negative control) and C2C12 cells (a skeletal muscle cell line known to express Ca_v_1.1 channels) (Fig 1B). Moreover, we previously showed that Ca_v_1.1 channels are expressed in the plasma membrane of CD4^+^ T cells [30]. We found that T cells express Ca_v_1.1 channels of identical molecular weight (180KDa) and at comparable levels to the C2C12 skeletal muscle cell line. Altogether, these data demonstrate that Ca_v_1.1 channels are constitutively expressed by CD4^+^ T lymphocytes. In particular, these channels are probably required for activation and differentiation of CD4^+^ T lymphocytes, where they are highly expressed.

### T Cells Express an Alternatively Spliced Variant of Ca_v_1.1 Channels with Deletion of Extracellular Linker Region, S3-S4 in Domain 4

**Fig 2:**
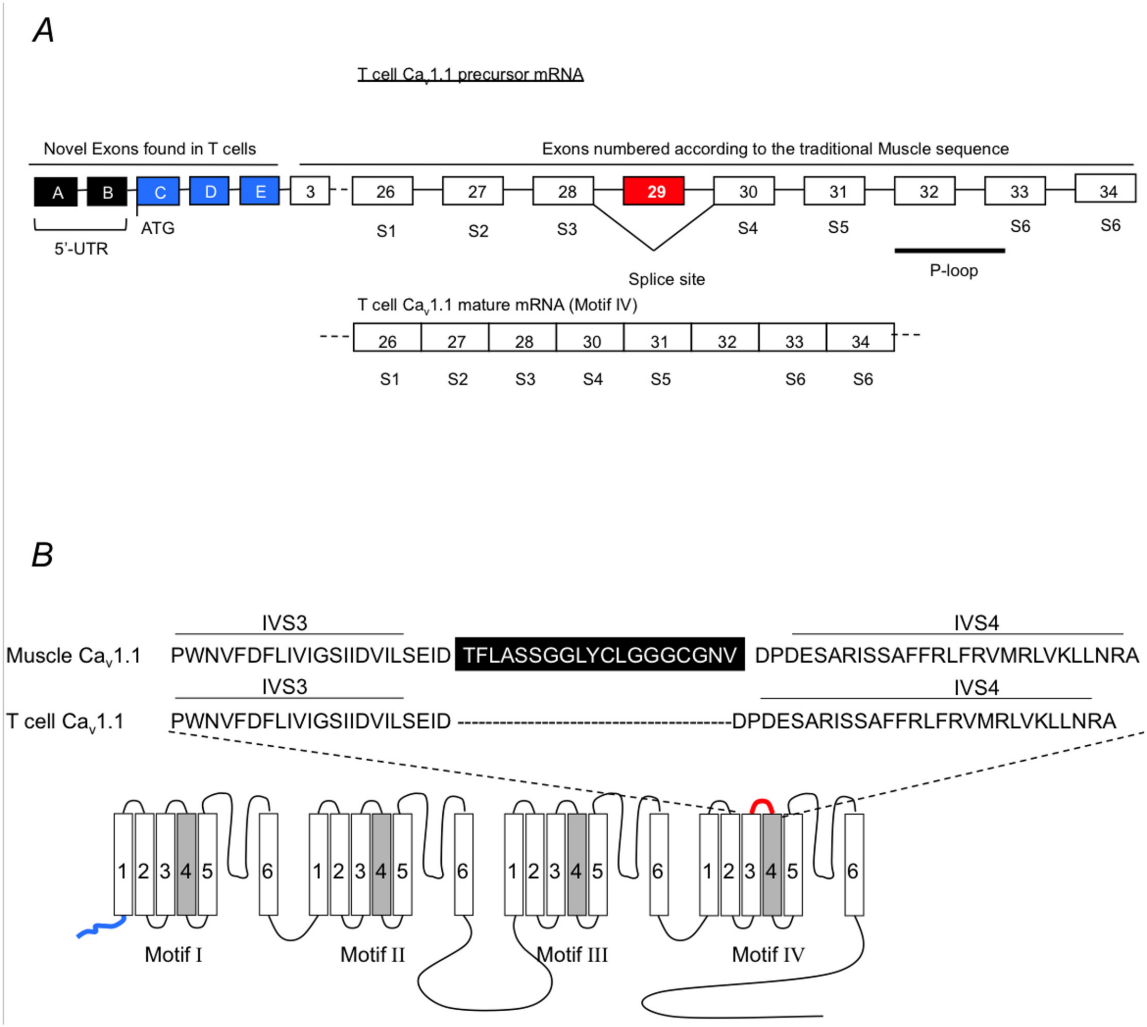
Schematic representation of mRNA splice sites and putative protein topology of the Ca_v_1.1 channel T cell variants. (A) Five new Exons were detected by 5’-RACE (A-E). The ATG of Ca_v_1.1 open reading frame is located in Exon C. Exons C-E replace the original Exons 1 and 2 (NM_000069). Alternative splice out of Exon 29 leads to the deletion of the IVS3-S4 linker. Exons encoding transmembrane segments are written below their respective boxes. Introns are represented as lines in between exon boxes. Below is a diagram of putative channel topology of the Ca_v_1.1 channel variant. (B) Diagram of putative channel topology of the Ca_v_1.1 variant. Transmembrane segments are white boxes, except the S4 voltage sensor domain, which is shown in gray. Sequence comparison between muscle and T cell Ca_v_1.1 at the IVS3-S4 linker region is shown.

We hypothesized that modifications might exist in T cells that affect this channel gating. By immunoblot analysis, we found that the molecular weight of Ca_v_1.1 channels was identical between muscle cells and T cells (Fig 1B). This suggests that only minor modifications occurred that affect the function of these channels in T cells. Since very little is known about the molecular structure of the Ca_v_1.1 channels in lymphocytes, we sequenced the full-length Ca_v_1.1 mRNA from DO11.10 mouse and Jurkat human T cells. To enable this, polymerase chain reaction (PCR) and 5’ RACE primers were designed, based on the muscle sequence (NM_000069) in order to isolate the Ca_v_1.1 α1 subunit cDNA from T cells. cDNA sequence analysis showed that the mouse and human sequences of the T cell and muscle Ca_v_1.1 channels are identical, except that a novel splice variant of the Ca_v_1.1 channels was expressed in T cells, which contained new exons, A through E, which replaced the original exons 1 and 2 of the muscle cells, and lacked exon 29 (57bps) due to alternative splicing (Fig 2A and 2B). Expression of exon 29 could not be detected in DO11.10, Jurkat cell lines, or primary C57BL/6 mouse spleen T cell cDNAs (not shown). The excision of exon 29 causes the deletion of the S3-S4 linker in motif IV of the T cell Ca_v_1.1 channels based on the published sequence (Fig 2B).

### Over Expression Analysis of T cell Ca_v_1.1 Channels in Non-Excitable Cells

Similar Ca_v_1.1 channel variants, lacking exon 29, were previously detected in muscle cells, where a modified voltage sensor activity was observed [37, 38]. However, compared to this muscle variant, the T cell variant contains five additional alternatively spliced exons (A-E) in the N-terminus of the channel, as reported above.

**Fig 3:**
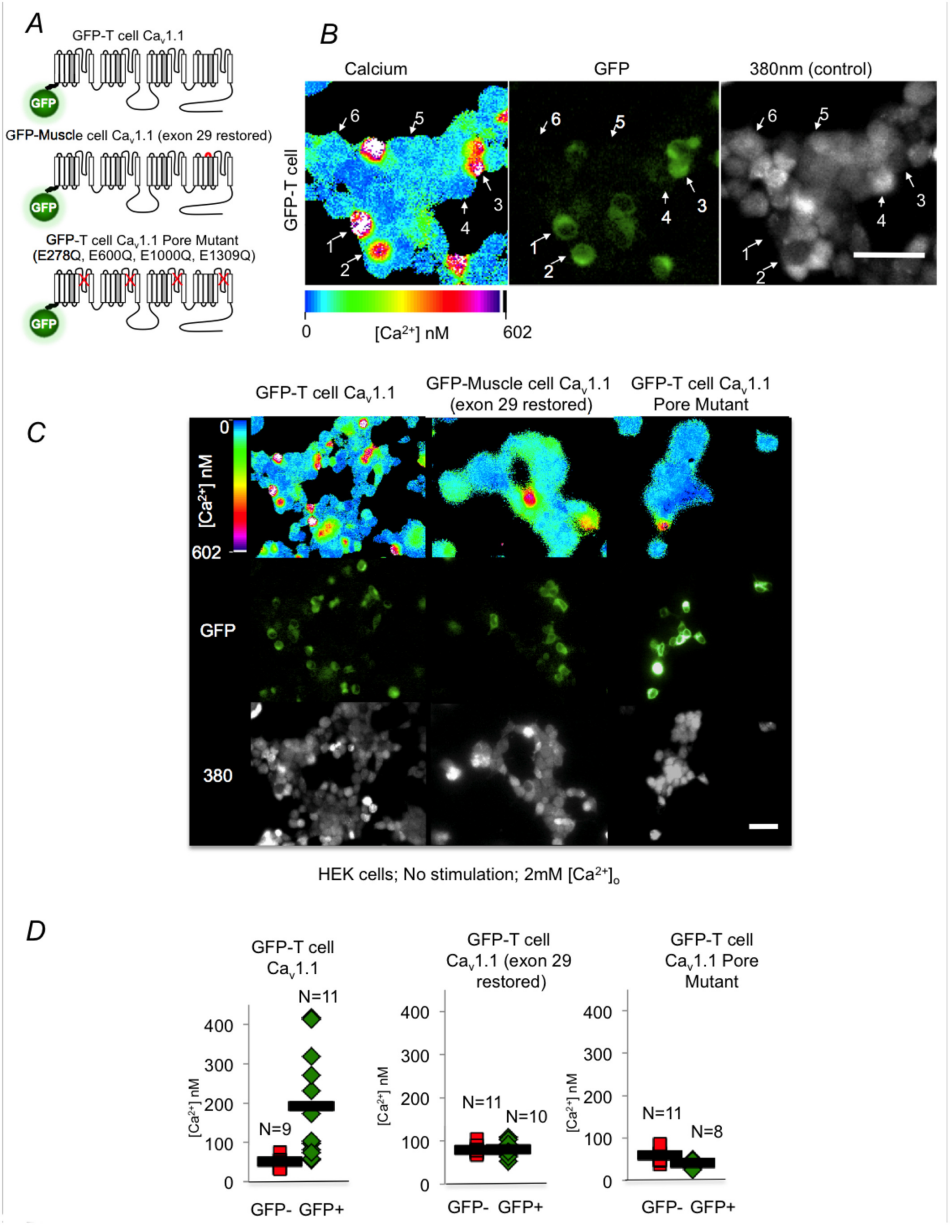
Calcium influx measurements through over expressed T cell Ca_v_1.1 channels. (A) Schematic representation of wild type T cell Ca_v_1.1 channel, a mutated construct containing insertion of Exon 29 and a pore-mutant. (B) HEK cells transfected with T cell Ca_v_1.1 construct described in A, were loaded with Fura-2 AM and Dual wavelength imaging was performed using InCyt Imaging system. A zoomed-in microscope picture montage is shown. Calcium concentration is shown in the left panel followed by the corresponding GFP and 380nm (the later is aimed at showing the entire population of cells in the picture). (C) HEK cells transfected with either of the constructs described in A, were loaded with Fura-2 AM and Dual wavelength imaging was performed using InCyt Imaging system. Calcium concentration is shown in the top panel and below are corresponding GFP and 380nm (the later is aimed at showing the entire population of cells in the picture). (D) Graphic representation of similar independent experiments as described in B and C. Results are representative of 5 independent experiments. *pValue = 0.003. 40X objective was used for all the recordings. Scale bar (white line located at the bottom right montage in B and C, denotes 50μ for all the montages presented.

To test the function of this Ca_v_1.1 channel variant in non-excitable cells, we cloned the Ca_v_1.1 channel cDNA from T cells and fused a green fluorescent protein to its N-terminus (Fig 3A; Henceforth designated as T cell Ca_v_1.1). This clone lacks exon 29 but contains exons A-E. Surprisingly, over-expression of these channels in Jurkat T cells was lethal, thus no GFP positive cells were detected. Instead, we expressed these channels in Human Embryonic Kidney cells (HEK), which proved to be more resilient to over-expression. GFP positive cells were detected within 24 hrs post transfection of T cell Ca_v_1.1 channels and cell death was observed within 48 hrs. Resting intracellular calcium levels were then measured in these transfected HEK cells using an InCyt dual wavelength ratiometric fluorescence imaging system and Fura-2 AM. This system allows gating on GFP positive cells while measuring their calcium content simultaneously. At 24 hours post transfection, we found that high GFP expression is correlated with high intracellular calcium levels. Therefore increasing expression of T cell Ca_v_1.1 channels results in increasing levels of intracellular calcium (Fig 3B- zoomed pictures; compare GFP positive cells 1–3 and GFP negative cells 4–6; see a broader picture in 3C- left column and an analysis graph in 3D- left graph). This positive correlation between intracellular calcium content and GFP expression suggests that these channels are constitutively open at resting potential, which is probably the underlying cause for the lethality of Ca_v_1.1 over expression in these cells.

To test if indeed the transfected Ca_v_1.1 channels conduct the calcium leading to the high intracellular calcium levels, we transfected T cell Ca_v_1.1 channels containing restored exon 29. Indeed, in this case, transfected GFP positive cells show low intracellular calcium levels similar to control GFP negative cells in the dish (Fig 3C and 3D). This suggests that restoration of exon 29 probably restored the gating activity that closes the pore at rest, thereby preventing cell death. In addition, this observation suggests that Ca_v_1.1 channels probably directly transmit calcium through the T cell membrane.

To further test the conductance of T cell Ca_v_1.1 channels, we mutated the channels pore (Glutamates of the P loops -E278Q, E600Q, E1000Q, E1309Q). Such pore mutations, where negatively charged glutamic acids are replaced with neutral glutamines, are expected to abrogate the calcium conductance of any calcium channels. Cells expressing these mutant channels show low intracellular calcium levels compared to control GFP negative cells in the dish (Fig 3C and 3D), consistent with the notion that these mutant channels do not constitutively conduct calcium. In conclusion, T cell Ca_v_1.1 channels (lacking exon 29) are likely to conduct calcium themselves.

**Fig 4:**
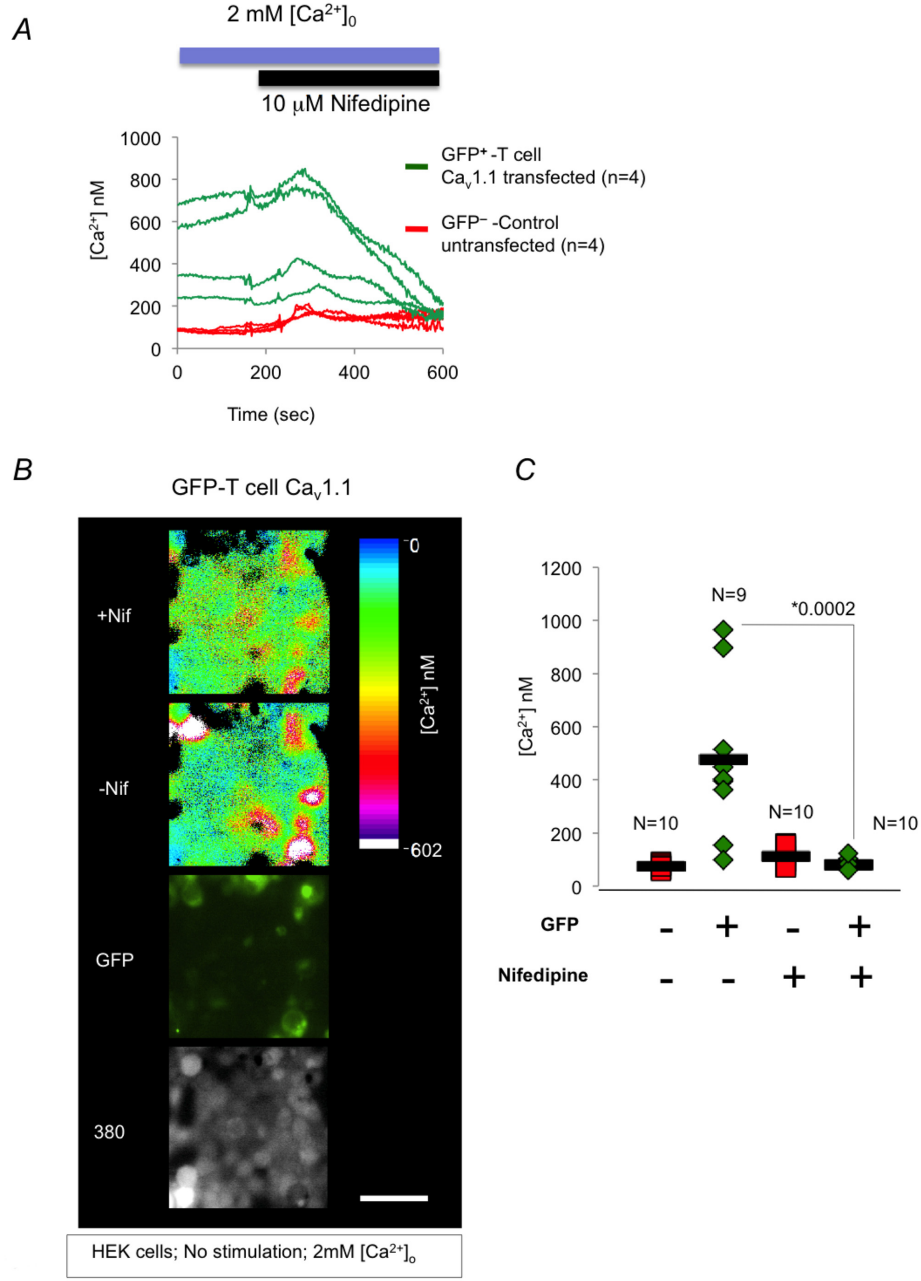
Effects of Nifedipine on calcium flow through T cell Ca_v_1.1 channels. HEK cells transfected with T cell Ca_v_1.1 channel plasmid construct, were loaded with Fura-2 AM and Dual wavelength imaging was performed using InCyt Imaging system. Nifedipine (10μM) was then added (indicated in A). Montages (B) or graphic representation (C) of calcium concentration is shown, before and after addition of nifedipine as described in Fig 3. *pValue = 0.0002 between GFP positive cells treated and untreated with nifedipine. 40X objective was used for all the recordings. The scale bar (white line located at the bottom right montage in B) denotes 50μ for all the montages presented.

Lastly, the addition of the Ca_v_ channel blocker- nifedipine to the medium of HEK cells transfected with T cell Ca_v_1.1 channels, significantly reduced calcium flux through T cell Ca_v_1.1 channels (Fig 4). Taken together, the evidence above demonstrates that calcium is directly transmitted through T cell Ca_v_1.1 channels in non-excitable cells.

As in Fig 3, the results presented in the montages and graph are independent experimental repeats. Results are representative of 5 independent experiments.

### TCR Induced Calcium Entry Is Dependent on Ca_v_1.1 Channel

Previously, we reported the expression pattern for Ca_v_1.1 channel mRNA, as well as other Ca_v_1 calcium channel family members, in primary T cells [27]. These studies showed that during primary stimulation of T cells, Ca_v_1.1 channel mRNA is constitutively expressed, even in naïve CD4 T cells [30]. Other studies in our lab have also shown that Ca_v_1.1 channels are critical for TCR induced calcium entry into CD4^+^ T cells. First, we showed that loss of function of Ca_v_1 β4 subunits results in reduced expression of Ca_v_1.1 channel expression, deficient calcium influx after TCR cross-linking and subsequent impairment in CD4^+^ T cell activation [27]. Second, we showed that loss of function of AHNAK1, a scaffold protein, which binds β subunits in CD4^+^ T cells, results in reduced plasma membrane expression of Ca_v_1.1 channels, reduced calcium influx after TCR cross-linking and subsequent low proliferation and IL-2 production [30].

**Fig 5:**
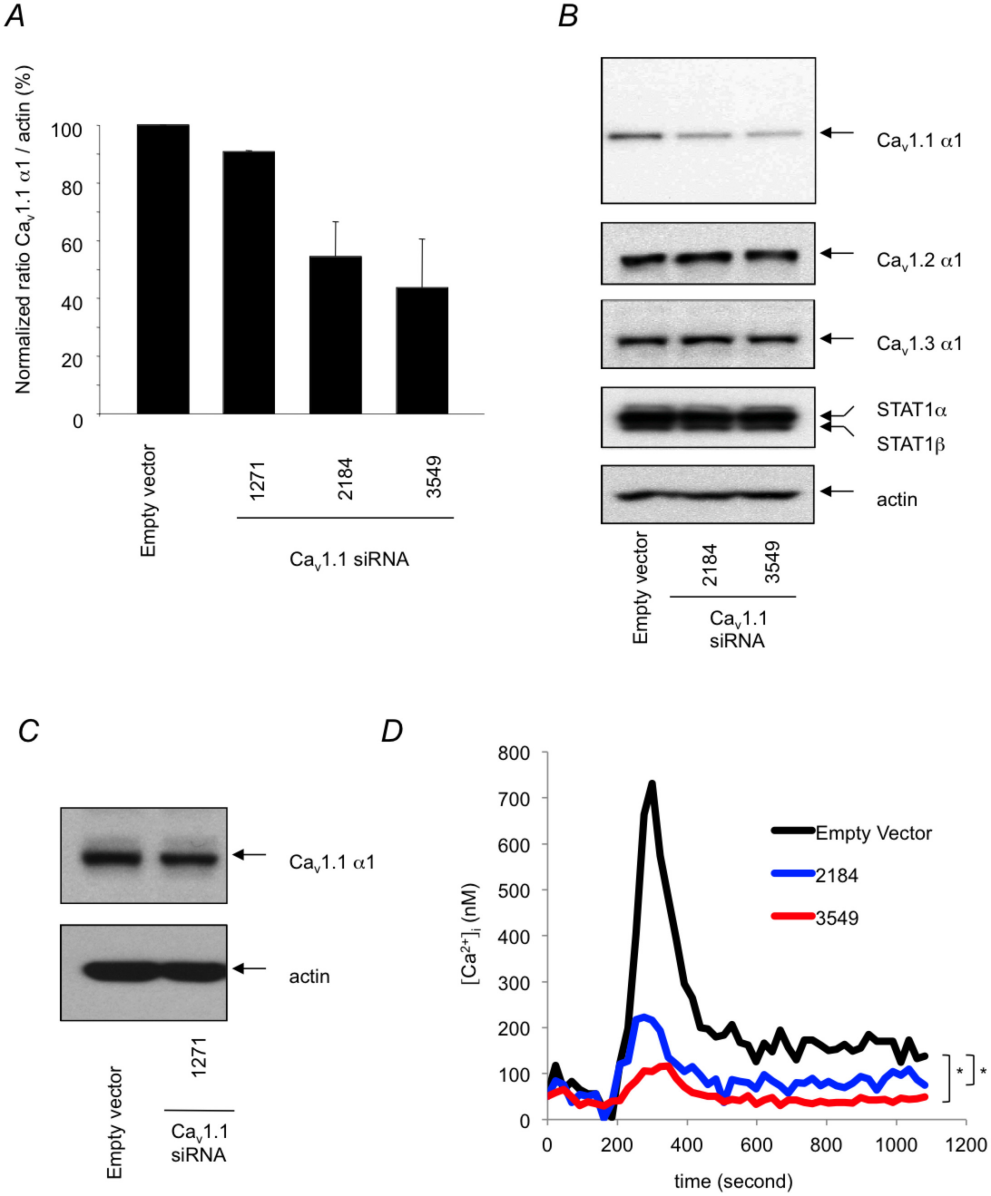
Characterization of calcium influx in Ca_v_1.1 knockdown T cells. (A) DO11.10 cells were transduced with lentivirus encoding Ca_v_1.1 1271 siRNA (1271), Ca_v_1.1 2184 siRNA (2184), Ca_v_1.1 3549 siRNA (3549), or with control virus containing pLL3.7 vector (empty vector). Three days later, GFP^+^ cells were sorted then Ca_v_1.1 α1 expression was assessed by immunoblot. Densitometry of Ca_v_1.1 α1 protein expression normalized to actin after background subtraction. Densitometry indicates mean + SD analysis of two independent experiments. (B) The expression of other genes was also tested to ensure specificity of siRNA activity. (C) A representative immunoblot used for Ca_v_1.1 1271 siRNA (1271) densitometry is shown. (D) Population based intracellular free calcium measurement in control (black line) and Ca_v_1.1 knockdown cells, 2184 (blue line), 3549 (red line) using ratiometric Fura2/AM calcium probe. Cells were stimulated by using a TCR cross-linking system with goat anti-hamster (GAH) Ab in calcium containing media. Calculation of the absolute calcium concentration was performed by normalizing to ionomycin response of each cell type. Results are representative of three independent experiments shown in S2 Fig. * = Statistically significant results. pValues are 1.0x10^-5^ and 2.3x10^-9^ for empty vector vs 2184 or 3549, respectively.

To study the functional role of the Ca_v_1.1 channel in T lymphocyte calcium entry, we used a lentivirus-based RNAi approach [39]. The DO11.10 T cell hybridoma was transduced with the appropriate viral preparations to knockdown Ca_v_1.1 gene expression. Subsequently, GFP^+^ cells were sorted and then tested for Ca_v_1.1 channel expression. We successfully reduced the expression of the Ca_v_1.1 gene using two independent siRNA sequences directed to different locations in the Ca_v_1.1 mRNA (Fig 5A and 5B) and thus named 2184 and 3549. We performed three independent viral transductions to generate knockdown cells using 2184 and 3549 siRNAs and similar inhibition of Ca_v_1.1 channel expression was observed. Immunoblot analysis revealed a typical and significant reduction of Ca_v_1.1 protein by 58% and 67% for 2184 and 3549 siRNAs, respectively (Fig 5A and 5B). Our negative control was a third sequence, named 1271, which yielded no inhibition of Ca_v_1.1 gene expression, suggesting that the inhibition by 2184 and 3549 was sequence specific (Fig 5A and 5C). In addition, the RNAi effect was specific to Ca_v_1.1 channel since the expression of other genes, including Ca_v_1.2, Ca_v_1.3 channels or STAT1α and STAT1β was unaffected (Fig 5B). Immunoblotting for actin was used to confirm that equal amounts of protein were loaded on the gel (Fig 5A).

We next investigated the role of Ca_v_1.1 channels in the sustained calcium influx following specific T cell activation. Using Fura-2/AM and a ratiometric calcium probe, we detected, under physiological calcium concentration conditions (~ 1 mM in the extracellular media), a significant reduction in the TCR-induced calcium response in both 2184 and 3549 Ca_v_1.1 channel knockdown cells relative to the control cells (Fig 5D and S2 Fig), which mounted a robust calcium response as shown by the absolute calcium concentration reached. Both the initial peak response and the plateau were inhibited (Fig 5D and S2 Fig). Interestingly, both 2184 and 3549 siRNA Ca_v_1.1 channel knockdown cells showed normal release of calcium from intracellular stores in response to thapsigargin stimulation, an inhibitor of calcium-ATPase pumps that depletes intracellular stores [40–42], in a calcium free medium (S1A and S1B Fig, see w/o calcium). Similar results were seen when TCR stimulation was performed in calcium free medium (data not shown). Together, these observations, as well as the normal PLCγ1 activation following TCR cross-linking in Ca_v_1.1 channel knockdown cells (S1C Fig), indicate that calcium release from intracellular stores is independent and temporally distinct from that of Ca_v_1.1 channel activity.

## Discussion

We have previously observed in vivo that T cells from mice lacking β4 or AHNAK1 show reduced plasma membrane expression of Ca_v_1.1 channels and reduced calcium entry after activation [13, 27, 30]. In the present study, we show that, similar to their roles in other tissues, such as muscle, Ca_v_1.1 channels are required for transmission of calcium into T cells after activation.

The present study provides new evidence for the role of Ca_v_1.1 channels during calcium entry into stimulated T cells. These channels are detected in naïve T cells and their expression is highly upregulated in activated T cells (at day 4 after activation in vitro). This suggests that these channels are employed during the immune response of T cells. These variant Ca_v_1.1 channels lack an extracellular loop located adjacent to the S4 voltage sensor of the 4^th^ domain, and contain five new alternatively spliced N-terminal exons. We show that reduction in Ca_v_1.1 channel expression in T cells results in reduced calcium entry after TCR stimulation. Over expression of cloned T cell Ca_v_1.1 channels in HEK 293 cells, shows that calcium influx through these channels occurs at the normal resting potential of these cells, probably due to exon 29 deletion.

Previous studies have provided significant insights into the outcome of exon 29 deletion on Ca_v_1.1 channel function in muscle cells, where a modification of voltage-dependence was observed [37, 38]. Calcium currents through these variants showed a -30-mV left-shifted voltage dependence of activation and a substantially increased open probability, giving rise to an increased current density [38]. Thus, aberrant myopathic muscle cells containing Ca_v_1.1 channels lacking exon 29 have increased calcium conductance [37, 38]. Compared to muscle cells, which have membrane potentials of around (-90mV), T cells or HEK 293 cells, maintain a more positive resting potential of ~-60mV [43–45]. Thus, in T cells or 293 HEK cells, such Ca_v_1.1 channel variants would be expected to show even higher calcium conductance at rest. This may provide an explanation for the constitutive calcium influx through these channels at the resting potential of such non-excitable cells. Other, as yet uncharacterized factors may, however, also contribute to the increased calcium flux observed upon over expression of these Ca_v_1.1 channels in 293 HEK cells. In addition, further studies will determine the specific function of the alternatively spliced exons A through E, found, so far, only in T cells.

It is important to consider these results in the context of the large body of work on other calcium conductance pathways in lymphocytes, in particular the CRAC pathway. In lymphocytes, calcium entry through calcium release activated calcium (CRAC) channels is the primary mechanism that has been characterized to be responsible for the increase of free intracellular calcium, which is in turn necessary for cell activation and cytokine production. Two novel molecules, STIM1 and ORAI1, have been identified as important for the CRAC current in lymphocytes (reviewed in [14–16]). Both CD4^+^ and CD8^+^ T cells express all STIM (STIM1-2) and ORAI (ORAI1-3) family members [46]. However, CD4^+^ T cell deficient in ORAI1 show minimal effects on calcium entry or proliferation after TCR cross-linking [17, 18]. A recent report suggests that TRPV1 is required for the activation of CD4^+^ T cells [12].

Our studies on the components of the Ca_v_ complex identify an alternative path of calcium influx in response to TCR stimulation. When the Ca_v_1.1 channel membrane expression is reduced by either deficiency in AHNAK1 or β4 subunits there is a decrease in calcium entry even though the CRAC channel pathway remains intact [27, 30]. We therefore believe that multiple channels, including CRAC and Ca_v_, are probably involved in calcium entry into T cells. It is possible that these channels function sequentially or independently, and further studies are required to resolve this issue. One step towards this goal was shown recently when the interactions between STIM1 and Ca_v_1.2 channels were described, leading to silencing of the latter [33, 34].

Clearly it will be of great interest to further unravel the relative contributions of the CRAC/TRP currents and the Ca_v_1.1 current for calcium entry in T cells.

## Material and Methods

### Activation and Differentiation of CD4 T Cells In Vitro

CD4 T cells from spleens and/or lymph nodes were isolated from 6- to 8-week-old C57BL/6 mice purchased from the National Cancer Institute (Frederick, MD). CD4 cells were isolated by immunomagnetic negative selection using Abs against CD8, NK1.1, and MHC class II, followed by incubation with anti-mouse and anti-rat Ig-coated magnetic beads (PerSeptive Biosystems). Cells were cultured in Bruff’s medium containing 10% FCS and were stimulated for various days in the presence of plate-bound anti-CD3 and anti-CD28 Abs. Cells were then harvested for further analysis. To generate Th1 cells, 3.5 ng/ml of IL-12 (a gift from Wyeth Research) and 2 μg/ml of anti-IL-4 mAb 11B11 were added to the culture, whereas 1000 units/ml of IL-4 and 2 μg/ml of anti-IFNγ mAb XMG1.2 were added to induce Th2 cells. 20 units/ml of recombinant huIL-2 (a gift from Biogen, Cambridge, MA) were added in both conditions.

The Yale University institutional animal care and use committee has approved this study. All mice were cared for in accordance with protocols established by the institutional animal care and use committee at the Yale University animal facility.

### Antibodies

The anti-Ca_v_1.1 Ab used against the dihydropyridine receptor (DHPR) α1S (Santa Cruz Biotechnology, Santa Cruz, CA), was an affinity-purified goat polyclonal Ab, which interacts with Ca_v_1.1 α1 subunit from mouse, human, and rat origin. For usage in immunoblot, anti-Ca_v_1.1 Ab was biotinylated as previously described [27]. The following antibodies were also used in this study: Rabbit polyclonal Ab against calnexin was a gift from Dr. Cresswell P.; Rabbit polyclonal Ab against Ca_v_1.2 and rabbit polyclonal Ab against Ca_v_1.3 purchased from Alomone labs, Israel. Rabbit phospho-PLCγ1 (Tyr783), rabbit PLCγ1 Abs were purchased from Cell Signaling, Danvers, MA.

### Protein Extracts and Immunoblot Analysis

CD4 T cells (6 to 10 x 10^6^) were resuspended in 100 μl of hypotonic solution (10 mM HEPES pH 7.9, 10 mM KCl, and 0.1 mM EDTA) containing protease and phosphatase inhibitors (cocktail tablets, Roche Diagnostics, Indianapolis, IN), and incubated for 10 min on ice. Then NP40 was added at 1% and the cells were centrifuged at 5000 rpm for 5 min. The supernatant was then recovered.

For transcription factors, 10 μg protein of cytoplasmic or nuclear extracts were subjected to 8–12% sodium dodecyl sulfate-polyacrylamide gel electrophoresis (SDS-PAGE), transferred to polyvinylidene difluoride membrane (Millipore, Bedford, MA), and immunoblotted with Abs followed by the appropriate horseradish-peroxidase-conjugated secondary antibodies. Immunoblots were visualized by chemiluminescence (SuperSignal, Pierce, Rockford, Illinois). For Ca_v_1.1 detection, 30 μg of protein extracts were electrophoresed on 6% or 3–8% SDS-PAGE gels.

### Analysis of Intracellular Calcium Concentration

**Population based assay:** Levels of intracellular calcium were measured using the ratiometric calcium-binding dye Fura-2/AM as previously described [27]. Cells were incubated with 5 μM of Fura-2/AM (Molecular Probes, Eugene, OR) for 30 min at 37°C. Subsequently, T cells were washed then incubated with Ab to CD3 for 30 min on ice. 50 μg/ml of anti-hamster IgG (GAH) was added at the indicated time to stimulate the cells by Ab cross-linking. Fluorescence was monitored in ratio mode using a fluorometer (Polarstar Galaxy, BMG labtechnologies, Offenburg, Germany). Collected data were analyzed using Fluostar Galaxy Software (BMG labtechnologies, Offenburg, Germany). Experiments were performed at room temperature (~20°C). At the end of each experiment, cells were treated with 5 μM ionomycin in calcium-containing medium, then with calcium-free medium supplemented with 5 mM of EGTA. Experimental 340/380 ratio were converted to [Ca^2+^]_i_ as previously described [27] according to the equation described by Tsien [47].

**Single cell based assay:** Intracellular calcium concentration was measured with a ratiometric dye Fura-2, as previously described [34]. Coverslips with cells grown on them were placed in DMEM Media containing 2 μM fura-2/acetoxymethylester for 45 min at 20°C. Cells were then washed with the same medium and fura-2 trapped inside cells was allowed to de-esterify for 30 min at 20°C. Cells were then placed in a solution containing: (mM) 130 NaCl, 5 KCl, 1 MgCl2, 2 CaCl2, 30 glucose and 25 Hepes-NaOH, pH = 7.2 and Calcium measurements were performed on an InCyt dual-wavelength fluorescence imaging system (Intracellular Imaging Inc.). Briefly, Fura-2 was excited at 340 and 380 nm (OMEGA Optical, XF1093, 1094 respectively) Intracellular calcium concentrations are represented by the ratio of the fluorescence intensities excited at 340 nm and 380 nm from groups of single cells. Data are shown of traces from groups of individual cells (~10–15 cells each), and are representative of three or more independent experiments.

### Lentivirus-Based RNA Interference

RNAi was performed as previously described [39]. Briefly, Lentivirus was produced by co-transfecting pLL3.7 and packaging vectors into 293FT cells. The supernatant was collected 48 h later. DO11.10 T cell hybridomas were supplemented with lentiviral particles that have been preincubated with 4 μl of lipofectamine (Invitrogen, Carlsbad, California) per ml of viral preparation for 20 min on ice. The cells were then spun at 2000 r.p.m. for 1 h at 30°C in a Beckman J-6M centrifuge. Supernatant was removed after infection and replaced with supplemented RPMI growth medium. DO11.10 cells were collected for experiments 72 h later. GFP^+^ cells were sorted on a FACSVantage (Becton Dickinson, San-Diego). The following siRNA sequences were identified using the CACNA1S (Ca_v_1.1) cDNA (Accesion number: XM_358335) and were used to silence the Ca_v_1.1 α1 gene:

Ca_v_1.1 1271 siRNA: AGTGCCATGACCTAGTGAA (bp 1271 to bp 1289)
Ca_v_1.1 2184 siRNA: CGTTAATGAGGTGAAAGAC (bp 2184 to bp 2202)
Ca_v_1.1 3549 siRNA: TGTGTTTGACTTCCTAATC (bp 3549 to bp 3567)

Sequencing and mutations of T cell Ca_v_1.1

Ca_v_1.1 sequence was performed using PCR based on the available sequence in the NIH database. The primers used are as follows:

5’ UTR forward: 5'-TGATGATTGCGGCCGCATGGAGCCGCCCTCACCCCAGGACGAG-3'
Exon 9 reverse: 5’ TCAAGTCGTCCACGTCCAT-3’
Exon 9 forward: 5’ ACCTCCGTGGTTACATGAGC-3’
Exon 18 reverse: 5’ TGTTGGTGGGACTGAAGATG-3’
Exon 12 forward: 5’ GACCCTCTTCACCATCGAAA-3’
Exon 28 reverse: 5’ TGGGTTTTTGGGGATGTAAC,
Exon 25 forward 5’ GGAAAGTTCTACAGCTGCAATG-3’
Exon 40 reverse: 5’ ATTGGAGGGATGACTTGGTC-3’
Exon 39 forward: 5’ CGATGGCACAGTCACCTTTA-3’
3’ UTR reverse: 5’ TCCAGCACTTTCTGGCTTTT-3’

PCR fragments from independent reactions were cloned (using PCR Topo-Invitrogen) and sequenced multiple times.

5' RACE: Invitrogen's 5'RACE system was used according to the manufacturer's protocol, catalog number (18374–058).

The primers used for the 5' RACE PCR were:

Primer 1: TGAAGTCCAGCACATTCCAGCCACTG
Primer 2: AGCGTCCTGGTGGAATAAGAAGCCGTAG

Human Ca_v_1.1 sequence NM_000069 was used for analysis. Ca_v_1.1 is located on chromosome 1q32. The accession number for the human contig that contains the Ca_v_1.1 genomic sequence is: NT_029862. In Jurkat cells, Muscle Exons 1 and 2 were replaced with three new exons (C-E). Ca_v_1.1 5’UTR located in Exon A and B and the new open reading frame starts at Exon C.

The new protein sequence is:

MPVPGHDVEAYCLLCECRYEERSTTTIKVIIVIYLSVVGALLLYMAFLMLVDPLIRKPDAYTEQLHNEEENE

This new structure replaces exons 1and 2 of the currently known form:

MEPSSPQDEGLRKKQPKKPVPEILPRPPRALFCLTLENPLRKACISIVEWKPFETIILLTIFANCVALAVYLPMPEDDNNSLNLGLEKLEYFFLIVFSIEAAMKII

Mutations in the P-loops of Ca_v_1.1 (E278Q, E600Q, E1000Q, E1309Q) were performed using Quick change Site Directed Mutagenesis kit (Stratagene). The primers used are as follows:

P-LOOP1F CAA TGC ATC TCC ATG CAG GGG TGG ACT GAT GTC
P-LOOP1R GAC ATC AGT CCA CCC CTG CAT GGA GAT GCA TTG
P-LOOP2F CAG GTC CTG ACG GGT CAA GAC TGG AAC TCT GTG
P-LOOP2R CAC AGA GTT CCA GTC TTG ACC CGT CAG GAC CTG
P-LOOP3F ACG GTC TCC ACC TTC CAG GGT TGG CCT CAG CTA
P-LOOP3R TAG CTG AGG CCA ACC CTG GAA GGT GGA GAC CGT
P-LOOP4F AGG TGT GCC ACA GGG CAA GCC TGG CAA GAG ATC
P-LOOP4R GAT CTC TTG CCA GGC TTG CCC TGT GGC ACA CCT

For the insertion of Exon 29 into the T cell Ca_v_1.1 we used the Quick Change site directed mutagenesis kit with the following primers:

INS57NTA3609F: TCA TCC TGA GCG AGA TCG ATA CTT TCC TGG CCT CCA GCG GGG GAC TGTATT GCC TGG GTG GAG GCT GCG GGA ACG TTG ACC CAG ATG
INS57NTA3609R: TCA TCT GGG TCA ACG TTC CCG CAG CCT CCA CCC AGG CAA TAC AGT CCCCCG CTG GAG GCC AGG AAA GTA TCG ATC TCG CTC AGG ATG A

## Supporting Information

S1 Fig Characterization of calcium release from intracellular stores in Ca_v_1.1 knockdown T cells. Fura-2 measurement of intracellular calcium for control (black line), 2184 (blue line) and 3549 (red line) Ca_v_1.1 channel knockdown cells following treatment by thapsigargin. Cells were stimulated in medium without calcium (w/o calcium) to deplete intracellular calcium stores, then followed by the addition of calcium (10 mM or 0.2 mM for (A) and (B) respectively) as indicated. A and B are representative of eight experiments performed for A and three for B. (C) PLCγ1 phosphorylation in control and Ca_v_1.1 knockdown cells was evaluated by immunoblot. Cells were left unstimulated or were stimulated for 2 min by TCR cross-linking before detection of total (PLCγ1) or phosphorylated PLCγ1 (p-PLCγ1). Same amounts of proteins were loaded and actin was used as an internal control and to ensure equal loading.
(TIF)

S2 Fig Characterization of calcium influx in Ca_v_1.1 knockdown T cells. Population based intracellular free calcium measurement in control (black line) and Ca_v_1.1 knockdown cells, 2184 (blue line), 3549 (red line) using ratiometric Fura2/AM calcium probe. Cells were stimulated by using a TCR cross-linking system with goat anti-hamster (GAH) Ab in calcium containing media. Calculation of the absolute calcium concentration was performed by normalizing to ionomycin response of each cell type. These are two additional individual experiments to Fig 5D. * = Statistically significant results. pValues are: for A: 2.0x10^-5^ and 5.5x10^-8^ for empty vector vs 2184 or 3549, respectively; For B: 6.8x10^-13^ and 1.1x10^-16^ for empty vector vs 2184 or 3549, respectively.
(TIF)
