# Supplementary figures and images for: T Cell Receptor Mediated Calcium Entry Requires Alternatively Spliced Cav1.1 Channels
Source: PLoS One. 2016 Jan 27;11(1):e0147379. doi: 10.1371/journal.pone.0147379 (PMC4729531; doi:10.1371/journal.pone.0147379)

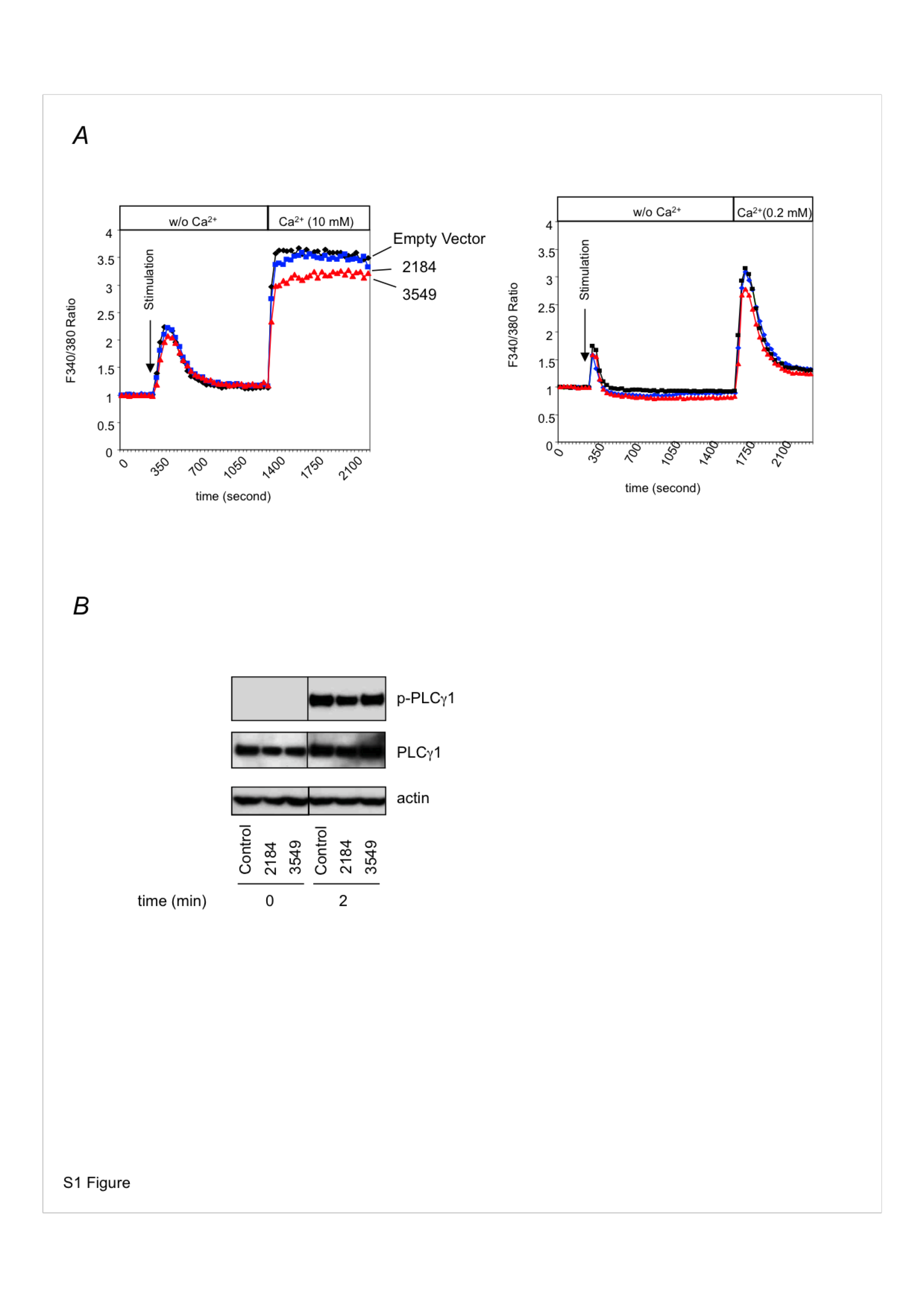

Supplement: S1 Fig — Fura-2 measurement of intracellular calcium for control (black line), 2184 (blue line) and 3549 (red line) Cav1.1 channel knockdown cells following treatment by thapsigargin. Cells were stimulated in medium without calcium (w/o calcium) to deplete intracellular calcium stores, then followed by the addition of calcium (10 mM or 0.2 mM for (A) and (B) respectively) as indicated. A and B are representative of eight experiments performed for A and three for B. (C) PLCγ1 phosphorylation in control and Cav1.1 knockdown cells was evaluated by immunoblot. Cells were left unstimulated or were stimulated for 2 min by TCR cross-linking before detection of total (PLCγ1) or phosphorylated PLCγ1 (p-PLCγ1). Same amounts of proteins were loaded and actin was used as an internal control and to ensure equal loading. (TIF) [file pone.0147379.s001.tif]

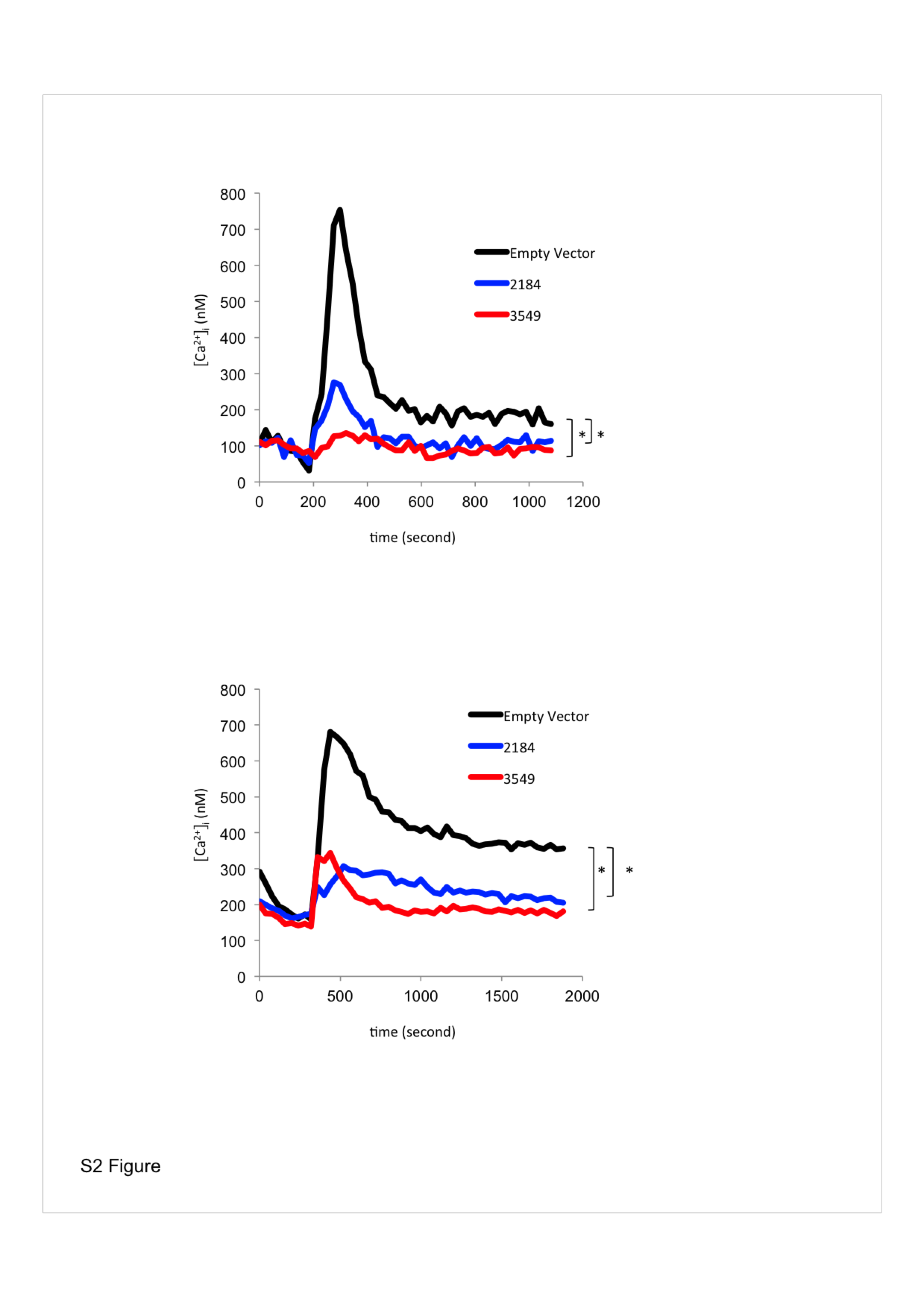

Supplement: S2 Fig — Population based intracellular free calcium measurement in control (black line) and Cav1.1 knockdown cells, 2184 (blue line), 3549 (red line) using ratiometric Fura2/AM calcium probe. Cells were stimulated by using a TCR cross-linking system with goat anti-hamster (GAH) Ab in calcium containing media. Calculation of the absolute calcium concentration was performed by normalizing to ionomycin response of each cell type. These are two additional individual experiments to Fig 5D. * = Statistically significant results. pValues are: for A: 2.0x10^-5^ and 5.5x10^-8^ for empty vector vs 2184 or 3549, respectively; For B: 6.8x10^-13^ and 1.1x10^-16^ for empty vector vs 2184 or 3549, respectively. (TIF) [file pone.0147379.s002.tif]
